# Inflammasome Genetic Variants Are Associated with Protection to Clinical Severity of COVID-19 among Patients from Rio de Janeiro, Brazil

**DOI:** 10.1155/2022/9082455

**Published:** 2022-09-05

**Authors:** Nathalia Beatriz Ramos de Sá, Milena Neira-Goulart, Marcelo Ribeiro-Alves, Hugo Perazzo, Kim Mattos Geraldo, Maria Pia Diniz Ribeiro, Sandra Wagner Cardoso, Beatriz Grinsztejn, Valdiléa G. Veloso, Artur Capão, Marilda Mendonça Siqueira, Ohanna Cavalcanti de Lima Bezerra, Cristiana Couto Garcia, Larissa Rodrigues Gomes, Andressa da Silva Cazote, Dalziza Victalina de Almeida, Carmem Beatriz Wagner Giacoia-Gripp, Fernanda Heloise Côrtes, Mariza Gonçalves Morgado

**Affiliations:** ^1^Laboratory of AIDS & Molecular Immunology, Oswaldo Cruz Institute, FIOCRUZ, Rio de Janeiro, Brazil; ^2^Laboratory of Clinical Research on STD/AIDS, National Institute of Infectology Evandro Chagas, FIOCRUZ, Rio de Janeiro, Brazil; ^3^Laboratory of Respiratory Virus and Measles, Oswaldo Cruz Institute, FIOCRUZ, Rio de Janeiro, Brazil; ^4^Center of Technological Development in Health (CDTS)/National Institute of Science and Technological for Innovation on Neglected Population Diseases (INCT-IDPN), FIOCRUZ, Rio de Janeiro, Brazil

## Abstract

COVID-19 has a broad spectrum of clinical manifestations, from asymptomatic or mild/moderate symptoms to severe symptoms and death. The mechanisms underlying its clinical evolution are still unclear. Upon SARS-CoV-2 infection, host factors, such as the inflammasome system, are activated by the presence of the virus inside host cells. The search for COVID-19 risk factors is of relevance for clinical management. In this study, we investigated the impact of inflammasome single-nucleotide polymorphisms (SNPs) in SARS-CoV-2-infected individuals with distinct severity profiles at clinical presentation. Patients were divided into two groups according to disease severity at clinical presentation based on the WHO Clinical Progression Scale. Group 1 included patients with mild/moderate disease (WHO < 6; *n* = 76), and group 2 included patients with severe/critical COVID-19 (WHO ≥ 6; *n* = 357). Inpatients with moderate to severe/critical profiles were recruited and followed-up at Hospital Center for COVID-19 Pandemic – National Institute of Infectology (INI)/FIOCRUZ, RJ, Brazil, from June 2020 to March 2021. Patients with mild disease were recruited at Oswaldo Cruz Institute (IOC)/FIOCRUZ, RJ, Brazil, in August 2020. Genotyping of 11 inflammasome SNPs was determined by real-time PCR. Protection and risk estimation were performed using unconditional logistic regression models. Significant differences in NLRP3 rs1539019 and CARD8 rs2043211 were observed between the two groups. Protection against disease severity was associated with the A/A genotype (OR_adj_ = 0.36; *P* = 0.032), allele A (OR_adj_ = 0.93; *P* = 0.010), or carrier-A (OR_adj_ = 0.45; *P* = 0.027) in the NLRP3 rs1539019 polymorphism; A/T genotype (OR_adj_ = 0.5; *P* = 0.045), allele T (OR_adj_ = 0.93; *P* = 0.018), or carrier-T (OR_adj_ = 0.48; *P* = 0.029) in the CARD8 rs2043211 polymorphism; and the A-C-G-C-C (OR_adj_ = 0.11; *P* = 0.018), A-C-G-C-G (OR_adj_ = 0.23; *P* = 0.003), C-C-G-C-C (OR_adj_ = 0.37; *P* = 0.021), and C-T-G-A-C (OR_adj_ = 0.04; *P* = 0.0473) in NLRP3 genetic haplotype variants. No significant associations were observed for the other polymorphisms. To the best of our knowledge, this is the first study demonstrating an association between CARD8 and NLRP3 inflammasome genetic variants and protection against COVID-19 severity, contributing to the discussion of the impact of inflammasomes on COVID-19 outcomes.

## 1. Introduction

At the end of 2019, a new disease emerged, described initially as an outbreak of viral pneumonia in individuals living in Wuhan, China [1]. Researchers identified a new coronavirus, severe acute respiratory syndrome coronavirus 2 (SARS-CoV-2), as the pathogen causing the outbreak *[*2*]*. The World Health Organization (WHO) named the associated coronavirus disease COVID-19 [3], which was raised to the pandemic category in March 2020 due to its fast dispersion around the world [4]. Globally, the mortality and incidence of SARS-CoV-2 have increased rapidly. Currently, the Americas are the continents most affected by the COVID-19 pandemic, and the United States of America (USA) and Brazil are the leaders in the numbers of cases to date [5]. According to official data from the WHO, more than 478 million individuals are already infected by SARS-CoV-2, and more than 6 million subjects have died due to COVID-19 worldwide [5, 6]. In Brazil, the first case was reported on February 26, 2020, and the first community transmission was identified on March 13, 2020. The country has accumulated more than 29 million reported cases, with more than 658,000 deaths, as of March 2022 [6]. SARS-CoV-2 vaccination started in January 2021 but was initially restricted to health care workers and elderly people. By April 2022, a total of 163 million individuals had been fully vaccinated, and more than 80 million subjects had received a booster vaccine dose, leading to a decrease in the incidence of severe disease and mortality [5].

The clinical presentation of COVID-19 can range from asymptomatic or mild/moderate flu-like symptoms to critical symptoms, such as severe acute respiratory syndrome (SARS), thromboembolism, sepsis, multiple organ failure, and death [6]. Although COVID-19 mortality rates vary among countries, older age and the presence of comorbidities have been strongly associated with more severe disease and death. The relationship between host genetics and the mechanisms underlying SARS-CoV-2 infection with the worst clinical evolution remains unclear [7–10].

In fact, during SARS-CoV-2 infection, host factors are activated by the presence of the virus inside host cells. Pattern recognition receptors (PRRs) recognize conserved virus fragments, known as pathogen-associated molecular patterns (PAMPs), and trigger the activation of several cellular components [11, 12]. Among the large family of PRRs are NOD-like receptors (NLRs), retinoic acid-inducible gene-I- (RIG-I-) like receptors (RLRs), and Toll-like receptors (TLRs) [13, 14]. Some studies have already shown that the RLR family is an important PRR in the detection of coronaviruses [15, 16]. In addition, NLR receptors stand out due to their wide recognition of intrinsic or extrinsic stimuli, operating principally as cytoplasmic sensors [17]. These receivers lead to activation of the NF-*κ*B signaling pathway, which culminates in the transcription of several molecules, such as gasdermin-D (GSDM-D), pro-IL-1*β*, and pro-IL-18, among others [18, 19]. These released molecules cause a wave of local inflammation involving increased secretion of proinflammatory cytokines and chemokines (e.g., IL-6, IFN-*γ*, CCL2, and CXCL10) [20, 21]. These and other cytokines have already been observed to be increased in SARS-CoV-2 infection, especially in more severe cases [20, 22].

The primary function of NLRs is to form a multiprotein complex known as the inflammasome. Inflammasomes are cytosolic multiprotein oligomers of the innate immune system that interact with several adapter proteins and are responsible for the activation of inflammatory responses, leading to activation of caspase-1 and inducing the release of the proinflammatory cytokines IL-1*β* and IL-18 [23]. Different PRRs (e.g., NLRP1 and NLRP3) can activate inflammasome assembly in response to specific stimuli, leading to inflammation and triggering the innate immune response. Inflammasome activation is strictly regulated by endogenous host proteins (e.g., CARD8 and HSP90) and by a variety of transcriptional and posttranscriptional mechanisms [24]. NLR inflammasomes comprise at least three components: a protein sensor (e.g., NLRP1 and NLRP3), an inflammatory caspase (e.g., caspase-1 and caspase-11), and an adapter molecule containing a CARD domain (e.g., ASC) [25]. In addition, twenty-two members of the NLR family have been described in humans and can be divided into four categories based on their functions: inflammasome formation, signal transduction, transcription activation, and autophagy [26]. The association between dysregulated inflammasome activity and the occurrence of certain human inflammatory diseases highlights the importance of this pathway in innate immune responses. As a regulatory mechanism, activation of inflammasome sensors (NLRP3 and AIM2) induces autophagy that subsequently impacts negatively the inflammasome function by inhibiting the formation and production of cytokines, such as IL1*β*, and degrading the inflammasome complex. Thus, autophagy accompanies inflammasome activation to limit inflammation by eliminating active inflammasomes [27]. As discussed in Sargazi et al., a potential correlation between SARS-CoV-2 and other coronavirus pathogens and autophagy has been suggested, indicating the relevance of targeting the autophagy pathway in the development of therapies for COVID-19 [28]. It has been shown that the SARS-CoV-2 proteins E, ORF3a, and ORF8b activate the NLRP3 inflammasome [17]. Mutations in inflammasome genes may lead to inflammatory disorders, such as chronic inflammation, autoimmunity, and viral infections [29–31]. For example, SNPs in the NLRP3 gene were found to be associated with a group of inflammatory disorders of genetic origin with exaggerated secretion of IL-1*β* [32].

Studies have already noted a relationship between inflammasome activation and COVID-19 *[*13, 33*–*36*]*. Inflammasome activation is one of the main theories to explain the cytokine storm that can occur during COVID-19, causing severe disease [13, 36]. NLRP3 activation in COVID-19 has already been described in tissues of COVID-19 patients. Additionally, higher levels of the inflammasome products IL-18 and Casp1p20 in COVID-19 patients were associated with severe disease [36]. Recently, it has been demonstrated that lung-resident macrophages infected with SARS-CoV-2 activate inflammasomes and release IL-1 and IL-18, leading to pyroptosis, which might contribute to lung inflammation [37]. However, data exploring the role of inflammasomes in SARS-CoV-2 infection remain scarce. Genetic factors contributing to the outcome of SARS-CoV-2 infection remain unclear; however, variants in specific sites of the ACE2 and TMPRSS2 genes, as well as the ABO locus, have already been considered genetic risk factors for COVID-19 outcomes [38–42]. Currently, new candidate genes have been described in the literature as influencing susceptibility to COVID-19. In this respect, Nia et al. showed that TNF*α*/TNF*β* polymorphisms might substantially affect COVID-19 susceptibility [43]. In a case-control study, Rokni et al. reported that carrying the A allele in TNFA-rs361525, the C allele in IL1RN-rs419598, and the A allele in IL6R-rs2228145 was related to susceptibility to developing COVID-19 [44]. These findings indicate that SNPs in several other candidate genes involved in the inflammatory response might also impact susceptibility to COVID-19. A recent study showed that two NLRP3 variants play an important role in severe and critical COVID-19 [45]. The search for risk and/or protection factors in severe COVID-19 is relevant for clinical management and deserves more investigation. Thus, in the present study, we investigated the impact of 11 single-nucleotide polymorphisms (SNPs) in NLRP3 rs10754558 (3′UTR), rs4612666 (intronic region), rs1539019 (intronic region), rs3806268 (exonic region), and rs35829419 (exonic region); CARD8 rs2043211 (exonic region) and rs6509365 (intronic region); AIM2 rs2276405 (intronic region); CASP-1 rs572687 (intronic region); IFI16 rs1101996 (intronic region); and IL-1*β* rs1143634 (exonic region) inflammasome genes in SARS-CoV-2-infected individuals at distinct severity stages at clinical presentation in a public reference center for COVID-19 in Rio de Janeiro, Brazil.

## 2. Materials and Methods

### 2.1. Study Design and Population

This is a case-control genetic study nested in the RECOVER-SUS study (NCT04807699), which is a prospective multicenter study that includes participants with SARS-CoV-2 infection who were hospitalized due to COVID-19 at “Instituto Nacional de Infectologia Evandro Chagas” of the “Fundação Oswaldo Cruz” (INI/FIOCRUZ). Patients 18 years or older with confirmed SARS-CoV-2 infection presenting moderate, severe, or critical COVID-19 profiles based on the WHO severity classification at clinical presentation were enrolled in the RECOVER-SUS cohort from June 2020 to March 2021. Details regarding patient eligibility, enrollment, inclusion/exclusion criteria, and the study design of the RECOVER-SUS clinical cohort study have been previously described [46]. For the present study, we analyzed a subset of the RECOVER-SUS cohort, including 451 patients who agreed to participate in the substudy, gave biological samples for the genetic analyses, and were recruited from June to October 2020 and from February to March 2021.

Additionally, a group with mild COVID-19 composed of 43 individuals 18 years or older with SARS-CoV-2 infection confirmed by RT-PCR with asymptomatic or mild disease severity (outpatients with COVID-19) were recruited in August 2020 by the Laboratory of Respiratory Virus and Measles – IOC/FIOCRUZ, Rio de Janeiro, Brazil, after disease resolution for blood collection.

For this study, both outpatients and hospitalized individuals without suspected, probable, or RT-PCR-confirmed SARS-CoV-2 infection, according to the WHO COVID-19 case definition, or those who did not sign the consent form were excluded.

This study was approved by the Ethics Committee of National Institute of Infectology Evandro Chagas (INI)/Oswaldo Cruz Foundation (FIOCRUZ), Rio de Janeiro, Brazil, under the approval number CAAE 32449420.4.1001.5262 and the Oswaldo Cruz Institute (IOC)/Oswaldo Cruz Foundation (FIOCRUZ), Rio de Janeiro, Brazil, under the approval number CAAE 68118417.6.0000.5248. All participants or their legal representatives signed an informed consent form prior to enrollment in the study. All methods were performed in accordance with the relevant guidelines and regulations.

Demographic and clinical data and blood samples were collected at the study entry visit (baseline). Skin color was self-declared following the classification system employed by the Brazilian Institute of Geography and Statistics (IBGE) [47]. IBGE is an entity linked to the Brazilian Federal Government and is responsible for collecting Brazilian statistical, geographic, cartographic, geodetic, and environmental information.

### 2.2. Clinical Profiles at Presentation

Clinical presentation was defined as mild, moderate, severe, or critical COVID-19 according to the WHO severity classification [48]. The mild group (WHO < 4) included asymptomatic outpatients or those with mild symptoms, such as cough, chest pain, coryza, dyspnea, odynophagia, anosmia, ageusia, digestive symptoms, headache, and/or myalgia. The moderate group (WHO 4-5 classification) included hospitalized symptomatic patients with no need for oxygen therapy or oxygen by mask or nasal prong. The severe group (WHO 6-8 classification) included hospitalized patients requiring oxygen via NIV (noninvasive ventilation) or high flow, intubation, and mechanical ventilation pO_2_/FiO_2_ ≥ 150 or SpO_2_/FiO_2_ ≥ 200, mechanical ventilation pO_2_/FiO_2_ < 150 (SpO_2_/FiO_2_ < 200) or vasopressors. The critical group (WHO 9-10 classification) included patients requiring mechanical ventilation pO_2_/FiO_2_ < 150 and vasopressors, dialysis, or extracorporeal membrane oxygenation (ECMO) and patients who died.

### 2.3. Genomic DNA Extraction

DNA was extracted from whole blood using a QIAamp DNA Blood Mini Kit (Qiagen, Hilden, Nordrhein-Westfalen, Germany) following the manufacturer's instructions. The DNA concentration was determined using a NanoDrop 2000 spectrophotometer (Thermo Fisher Scientific, Waltham, Massachusetts, USA). The filtrates containing the isolated DNA were stored at -20°C until the genomics analyses.

### 2.4. Single-Nucleotide Polymorphism Selection and Genotyping

We selected 11 SNPs in six inflammasome genes [49–51], considering the relevance of each gene in the inflammasome pathway: CARD8 (rs2043211 and rs6509365), AIM2 (rs2276405), IFI16 (rs1101996), CASP-1 (rs572687), IL-1*β* (rs1143634), and NLRP3 (rs10754558, rs1539019, rs4612666, rs3806268, and rs35829419). SNP genotyping was performed using commercially available TaqMan assays (Applied Biosystems/AB and Life Technologies) according to the manufacturer's instructions (Applied Biosystems/AB and Life Technologies). Briefly, for qPCR, a final volume of 10 *μ*L was used, and 4.50 *μ*L of the genomic DNA, which was adjusted to 0.2 ng/*μ*L, was placed in each well of a 96-well fast plate with 5 *μ*L of the reaction buffer 2X TaqMan Master Mix and 0.5 *μ*L of the 20X Assay Working Stock of each gene target. Genotyping was conducted with a reaction of one cycle of 95°C for 10 min for polymerase activation, one cycle of 95°C for 15 seconds for denaturation, and 60°C for 1 min for annealing/extension in an ABI7500 Real-Time PCR platform. Allelic discrimination was carried out using Thermo Fisher Connect Software. The SNP characteristics are listed in Supplementary Table 1.

### 2.5. Statistical Analyses

For statistical analyses, we divided the COVID-19 patients into two groups according to WHO scores < 6 or ≥6. Group 1 included patients with mild and moderate COVID-19 (*n* = 76; WHO < 6), and group 2 included patients with severe and critical COVID-19 (*n* = 357; WHO ≥ 6).

The Mann–Whitney *U* tests were used to compare baseline demographic and clinical, continuous numerical variables, and Fisher's exact tests were used for categorical variables. In the SNP analyses, the frequencies of genotypes were determined by direct count, and *χ*^2^ tests assessed deviations from HWE. Pairwise LD patterns were determined for each gene using *r*^2^ statistics (cutoff of *r*^2^ ≥ 0.8). The homozygous genotypes of the allele with the major frequency in our sample were compared with the other genotypes including the minor allele frequency allele (carriers) to better observe the differences caused by the variation. The protection/risk estimate is presented as adjusted odds ratios (aORs) with a 95% CI for each SNP and estimated through unconditional logistic regression models. We included any clinical phenotypic marker associated with COVID-19 as a confounder in modeling all other genotypic analyses to eliminate any possible bias. Haplotype frequencies were estimated by maximum likelihood, and phase uncertainty was included in statistical models applied for association analyses. The most frequent haplotypes of the NLRP3 and CARD8 genes were considered references for the haplotype analyses. Multiple comparisons were corrected by estimations of false-discovery rates (FDRs). All statistical analyses were performed using R version 4.1.1 (R Core Team, 2021).

We post hoc estimated the statistical power of the study considering (a) the observed minor allele frequencies (MAFs) observed in our sample's controls (i.e., those with WHO < 6) for the studied gene variants (SNPs); (b) the prevalence of severe COVID-19 prevaccination cases of 36.17% previously reported for the Brazilian population in the same period [52]; and (c) the ratio between controls and cases of 1 : 4.7 (or 76/357). We conducted simulations assuming that gene variants were under a genetic additive model with a 95% association with the disease genotype, given by the D' linkage-disequilibrium measure. Simulations were conducted with R and packages “genetics” and “GeneticsDesign.”

## 3. Results

### 3.1. Sociodemographic and Clinical Characteristics

A total of 494 individuals with COVID-19 were included in this study. Of those, 61 patients with missing data needed for classification according to the WHO severity classification were excluded from the analysis. Thus, 433 individuals were included in this study and divided into two major groups according to the WHO severity classification, and their sociodemographic and clinical characteristics are depicted in Table 1. The mean age was 58 years (IQR = 21.79), with a mean of 50 years (IQR = 24.86) among the mild and moderate patients (WHO < 6 group) and 58 years (IQR = 22.64) among the severe and critical patients (WHO ≥ 6 group). Overall, 256 individuals (51.8%) were male, with 33 (43.4%) in the mild and moderate group and 189 (52.9%) in the severe and critical group. Regarding schooling, 145 (29.4%) of the individuals had a high school degree, with 15 (19.7%) in the mild and moderate group and 109 (30.5%) in the severe and critical group. The most common symptoms (>80%) in both groups were chest pain, diarrhea, abdominal pain, and nausea. In addition, the severe and critical groups presented a high frequency of (>80%) coryza, odynophagia, anosmia, loss of taste, headache, and myalgia as the most frequent symptoms. Most individuals included in this study (*n* = 323; 74.6%) were classified as having WHO severity scores between 6 and 8. The schooling, skin color, the comorbidity coronary artery disease, and some symptoms (fever, odynophagia, anosmia, loss of taste, and diarrhea) differed significantly between the groups. After correction by age, gender, skin color, schooling, diabetes mellitus, coronary artery disease, and obesity or previous bariatric disease comorbidities, wherever applicable, only oxygen supplementation or use of ventilatory support and a saturation level below 95% were significantly different between groups (Table 1).

### 3.2. Alleles, Genotypes, and Haplotype of Inflammasome Genes

#### 3.2.1. Association of Alleles and Genotypes between the Groups

The genotypes, alleles, and carrier frequencies of all the studied SNPs associated with disease severity protection or risk are shown in Table 2. Genotype frequencies associated with the 11 SNPs analyzed were in the Hardy-Weinberg equilibrium in both groups (Supplementary Table 1).

In this study, both NLRP3 rs1539019 and CARD8 rs2043211 polymorphisms were associated with protection against disease severity in SARS-CoV-2-infected individuals (Table 2). For NLRP3 rs1539019, the association was with carrying the A/A genotype (OR_adj_ = 0.36 [95% CI, 0.14-0.92], *P* = 0.033), allele A (OR_adj_ = 0.93 [95% CI, 0.88-0.98], *P* = 0.010), or carrier-A (OR_adj_ = 0.45 [95% CI, 0.22-0.91], *P* = 0.027). For CARD8 rs2043211, the association was with carrying the A/T genotype (OR_adj_ = 0.5 [95% CI, 0.25-0.99], *P* = 0.046), allele T (OR_adj_ = 0.93 [95% CI, 0.88-0.99], *P* = 0.018), or carrier-T (OR_adj_ = 0.48 [95% CI, 0.25-0.93], *P* = 0.029).

The frequency of the T allele in the NLRP3 rs4612666 polymorphism was slightly different between the group of patients with mild and moderate disease (29.61%) and those with severe to critical disease (38.52%) (OR_adj_ = 1.05 [95% CI, 1-1.11], *P* = 0.062). Additionally, the T/T genotype showed a frequency of 5.26% in the mild and moderate group and 16.81% in the severe and critical group, constituting a genetic marker with a trend for the risk of disease severity in SARS-CoV-2-infected individuals (OR_adj_ = 3.41 [95% CI, 0.93-12.59], *P* = 0.065).

The SNPs in CARD8 (rs6509365), IFI16 (rs1101996), CASP-1 (rs572687), IL-1*β* (rs1143634), AIM2 (rs2276405), and NLRP3 (rs3806268, rs35829419, and rs10754558) did not reveal any significant associations with disease severity risk and/or protection in SARS-CoV-2-infected individuals.

#### 3.2.2. Association of Haplotypes between the Groups

With respect to the NLRP3 genetic haplotype variants (rs1539019 - rs4612666 - rs3806268 - rs35829419 - rs10754558), carrying the A-C-G-C-G (OR_adj_ = 0.11 [95% CI, 0.02-0.69], *P* = 0.018), A-C-G-C-G (OR_adj_ = 0.23 [95% CI, 0.09-0.62], *P* = 0.004), C-C-G-C-C (OR_adj_ = 0.37 [95% CI, 0.16-0.86], *P* = 0.022), and/or C-T-G-A-C (OR_adj_ = 0.04 [95% CI, 0-0.96], *P* = 0.047) haplotypes were associated with protection against disease severity in SARS-CoV-2-infected individuals (Table 3). No haplotype of the CARD8 genetic variants was associated with risk and/or protection against disease severity in COVID-19 (Table 3). These analyses were performed considering the most frequent haplotype of the NLRP3 (C-T-G-C-C haplotype) and CARD8 (AA) genes as references.

Considering the observed minor allele frequencies (MAFs) of 0.02 (rs2276405/T and rs35829419/A), 0.19 (rs572687/A), 0.20 (rs1143634/A), 0.30 (rs4612666/T), 0.31 (rs1101996/C), 0.33 (rs2043211/T), 0.34 (rs6509365/G), 0.36 (rs3806268/A), 0.38 (rs10754558/G), and 0.45 (rs1539019/A) observed in our sample's controls (i.e., those with WHO < 6) for the studied gene variants (SNPs) and the prevalence of severe COVID-19 prevaccination cases of 36.17% previously reported for the Brazilian population in the same period [52], we estimated statistical powers for the study under a genetic additive model framework of 80% for MAFs between 0.19 and 0.34 to accept aORs ≥ 2.6 (or ≤0.38). For MAFs, between 0.36 and 0.45 was sufficient to accept aORs greater or equal to 2.8 (or ≤0.36) with equal estimated power. Only for the variant with an extremely low MAF, of 0.02, the sample size was insufficient. Indeed, we estimated 80% statistical power for this MAF only for aORs > 4 (<0.25).

### 3.3. Inflammasome Gene Polymorphisms and COVID-19-Associated Comorbidities

As the individuals included in the study had several comorbidities (Table 1), e.g., diabetes mellitus (DM), systemic arterial hypertension (SAH), coronary artery disease (CAD), and obesity or previous bariatric surgery (Ob), they were taken into consideration in all analyses between the groups (mild and moderate group vs. severe and critical group), as shown in the footnotes of all tables. Furthermore, to make sure that the results found were not influenced by the associated comorbidities, we performed an analysis with the general population of our study and the comorbidities present in the cohort.

The association of the major comorbidities identified in the COVID-19 individuals included in the present study with the inflammasome SNPs analyzed here is presented in Supplementary Tables 2S-4S. Briefly, carrier-A in the CARD8 rs2043211 polymorphisms (OR_adj_ = 0.17 [95% CI, 0.04-0.85], *P* = 0.031) was associated with protection against CAD (Table 2S). Similarly, protection against DM was associated with carrier-C (OR_adj_ = 0.47 [95% CI, 0.24-0.91], *P* = 0.024) in the NLRP3 rs1539019 polymorphism (Table 3S). Protection against obesity was associated with carrying the G/A genotype (OR_adj_ = 0.42 [95% CI, 0.23-0.78], *P* = 0.006) or carrier-A (OR_adj_ = 0.48 [95% CI, 0.27-0.85], *P* = 0.012) in the NLRP3 rs3806268 polymorphisms, whereas a slightly increased risk for obesity was observed for those carrying the A allele in the NLRP3 rs35829419 polymorphism (OR_adj_ = 1.21 [95% CI, 1.02-1.44], *P* = 0.029) (Table 4S). Concerning the analysis of the inflammasome haplotypes (Table 5S), carriers of the NLRP3 C-T-G-C-G haplotype had an increased risk for CAD (OR_adj_ = 11.82 [95% CI, 2.43-57.59], *P* = 0.002). No other association between comorbidities and inflammasome haplotypes was observed (data not shown). No significant associations between hypertension and the SNPs included in this study were observed (data not shown).

It is important to point out that none of the inflammasome polymorphisms found to be associated with CAD, DM, or obesity comorbidities showed any significant association when comparing the COVID-19 mild/moderate group with the severe/critical group (Tables 2 and 3).

## 4. Discussion

Innate immune receptors are essential in the sensing of infectious organisms, continuously monitoring the extracellular milieu as well as intracellular compartments. The inflammatory process in cells is often mediated by inflammasomes, which are cytosolic multiprotein oligomers of the innate immune system [53]. Inflammasomes tend to aggregate in response to various endogenous or exogenous stimuli and orchestrate the development of local and/or systemic inflammation [53, 54]. The mechanism of inflammasome activation in COVID-19 is still poorly explored. However, Rodrigues et al. showed that the NLRP3 inflammasome is activated in hospitalized patients infected with SARS-CoV-2 [36]. This suggests a role of the NLRP3 inflammasome in the pathophysiology of the disease, as a marker of disease severity and a potential therapeutic target for COVID-19. Toldo et al. identified the presence of inflammasomes in the lungs of patients with fatal COVID-19 [55]. On the other hand, several studies of the genes involved in assembling inflammasome complexes have attempted to explain their role in the heterogeneity of disease. For the same infection, some individuals are more susceptible to developing the disease, while others remain asymptomatic [25]. The present study demonstrated that genetically specific profiles (alleles, genotypes, and haplotypes) of NLRP3 rs1539019 and CARD8 rs2043211 polymorphisms were associated with protection against disease severity in SARS-CoV-2-infected individuals. Our data suggest that these SNPs might modulate inflammasome activation, contributing to protection against disease severity. Indeed, in a recent study [45], two other NLRP3 SNPs, NLRP3 rs10157379 and rs10754558 polymorphisms, were associated with an important role in severe acute respiratory syndrome (SARS) and severe and critical COVID-19 [45]. There was no significant association between NLRP3 rs10754558 and decreased COVID-19 severity risk or protection in our cohort. Although both studies included Brazilian individuals, our study was focused on people from the Southeast and North regions, whereas the study of Maes et al. included COVID-19 patients from one city in South Brazil, with a predominance of Caucasian ethnicity in their study group, while self-declared brown individuals predominated in our cohort. We do not know if this difference in ethnicity predominance between the two studies contributed to the differences in our results.

The NLRP3 rs1539019 polymorphism is an intronic variation whose function is still not entirely understood. However, several studies have reported that intronic polymorphisms may be associated with susceptibility/resistance to several diseases, such as rheumatoid arthritis [56], type II diabetes [57], and coronary artery disease [58]. One explanation for this is that many transcription factors bind to intronic sites that may play a role in regulating gene expression. In a study by Chung et al., the C allele of the NLRP3 rs1539019 polymorphism was found to be associated with the risk of renal cell carcinoma [59]. Additionally, Estfanous et al. reported that rs1539019 is associated with susceptibility to hepatitis C and a lower response to IFN treatment, depending on the allele and/or genotype. Moreover, Dehghan et al. reported a statistically significant association between the NLRP3 rs1539019 polymorphism and the risk of cardiovascular disease [60, 61]. To the best of our knowledge, our study is the first to demonstrate an association of the genotype A/A, allele A, or carrier-A in the NLRP3 rs1539019 variant with protection against disease severity in SARS-CoV-2-infected individuals. What is still unclear is the exact molecular mechanisms by which the NLRP3 rs1539019 polymorphism plays a protective effect in the outcome of COVID-19. It is possible that many transcription factors bind to intronic sites that may play a role in regulating gene expression [62]; therefore, we suggest that this polymorphism may involve an area containing a positive regulatory sequence. However, this hypothesis needs to be confirmed in further functional investigations.

The NLRP3 inflammasome, also known as NALP3 and cryopyrin, is currently the most studied inflammasome and is considered the main study model of these cytoplasmic complexes. The NLRP3 gene is located on the long arm of chromosome 1q44 and reacts to a diverse set of endogenous or exogenous stimuli [63]. NLRP3 has been linked to the pathogenesis of several diseases, including [1] metabolic disorders, such as type 2 diabetes [64], obesity [65], and autoimmune and inflammatory diseases [66–68]; neurological diseases [69]; and [2] diseases caused by viral pathogens, such as HIV [50], influenza A [70], and SARS-CoV [71]. SNPs in the *NLRP3* gene have already been associated with a group of inflammatory disorders of genetic origin [32, 72]. Other inflammasomes and molecules related to the activation cascade (e.g., CARD8, AIM2, IFI16, CASP-1, and IL-1*β*) have also been found to be associated with a variety of infections and metabolic diseases. They may affect the function of the NLRP3 inflammasome [73, 74]. From a functional perspective, the results of another study showed that SARS-CoV-2 upregulates the expression of genes involved in inflammatory processes, such as NLRP3, while downregulating the genes in the autophagic pathway [28].

Caspase recruitment domain–containing protein (CARD) 8 mediates inflammasome activation in response to various pathogen-associated signals [24]. CARD8 plays an important role in apoptosis regulation, inhibition of the activation of NF-*κ*B and caspase-1, and cytokine regulation [75]. CARD8 polymorphisms have been associated with several diseases, such as HIV-1 [76], ischemic stroke [77], and type 2 diabetes mellitus [78].

The CARD8 rs2043211 polymorphism has already been associated with risk and/or protection against several diseases, such as cardiovascular disease [79], atherosclerotic coronary artery disease [80], and inflammatory diseases, such as inflammatory bowel disease [81]. The rs2043211 variant of CARD8 is an A > T transversion on the template strand that introduces a premature stop codon, which results in the expression of a severely truncated CARD8 protein; therefore, this variant is unable to suppress NF-KB activity, which leads to high constitutive levels of pro-IL-1*β* [82]. In a recent study by our group, we verified an association between the CARD8 variant rs2043211 and protection against immune reconstitution inflammatory syndrome (IRIS) associated with HIV-TB coinfection (de Sá et al., 2022 unpublished data). Although COVID-19 has an important inflammatory profile [83] and constitutive increases in pro-IL-1*β* contribute to the cytokine storm that worsens the clinical status of patients [84], in our study, we found that the allele T, carrier-T, or genotype A/T in CARD8 rs2043211 polymorphisms is associated with protection against disease severity in individuals infected with SARS-CoV-2. One explanation for this is the interaction between CARD8 and NLRP3 [85]. Roberts et al. reported that a combination of CARD8 rs2043211 and NLRP3 rs35829419 has a protective effect against Crohn's disease, which is an inflammatory disease, by preventing the NLRP3 inflammasome from excessively producing interleukin-1*β* [85]. In our study, both CARD8 rs2043211 polymorphisms and NLRP3 rs1539019 polymorphisms had a protective effect against disease severity; thus, we hypothesized that this protective effect could be explained by their interaction, although the mechanism underlying this positive association with protection against disease severity in SARS-CoV-2 is not fully elucidated.

To the best of our knowledge, this is one of the first studies demonstrating an association between CARD8 genetic variants and protection against disease severity in SARS-CoV-2-infected individuals. Studies linking CARD8, NLRP3, and SARS-CoV-2 infection are still scarce due to the recent emergence of this pathogen. One recent study showed that the inflammasome is robustly activated in SARS-CoV-2-infected hospitalized individuals [36]. In addition, several studies have indicated that the inflammasome may be involved in the pathogenesis of the disease [13, 33–36].

In the present study, we classified patients at presentation according to the WHO severity classification and identified CARD8 and NLRP3 polymorphisms associated with protection against COVID-19 severity. No polymorphisms associated with a higher risk of disease severity were observed in our analyses.

Although selected inflammasome polymorphisms were associated with protection/susceptibility to some comorbidities observed in our study group (coronary artery disease, diabetes mellitus, and obesity), none of them showed a significant association when comparing the mild/moderate COVID-19 group with the severe/critical COVID-19 group. In our study, we found that the CARD8 rs2043211 polymorphism was associated with protection against coronary artery disease. Several studies have tried to demonstrate the role of this SNP in CAD, but no consistent association has been described thus far [80, 86, 87]. We also found that carrier-C in the NLRP3 rs1539019 polymorphism and the G/A genotype of the NLRP3 rs3806268 polymorphism were associated with protection against diabetes mellitus and obesity, respectively; on the other hand, carrying the A allele in the NLRP3 rs35829419 polymorphism was associated with a risk of obesity. To the best of our knowledge, this is the first time that these polymorphisms have been associated with these comorbidities.

Some limitations of the current study should be noted, mainly concerning the limited sample size of the mild/moderate group. Moreover, although the Brazilian population has an extensive mixture of ethnic/racial origins, the frequency of these and other SNPs is not consistent throughout the different populations in the world, justifying large further international studies or meta-analyses using already published data from different countries to assess the associations of genetic background with COVID-19 clinical profiles and outcomes. Future studies combining inflammasome genetic polymorphisms and functional analysis will be of foremost relevance to better understand the role of this cytoplasmic protein complex and its downstream effector inflammatory factors in the outcomes of SARS-CoV-2 infection.

Concerning the impact of the COVID-19 vaccination in the individuals analyzed in the present group, it is important to note that the participants included in 2020 were not vaccinated. For those recruited in 2021 (until March), we have no information on vaccination status [46]; however, the inpatients recruited in this period had WHO scores of 8-9, which eliminated any bias potentially caused by vaccination in the association between SNPs and disease protection observed in our study. Moreover, in Brazil, administration of the COVID-19 vaccine began in 2021 (end of January) exclusively for elderly people (>80 years) and health care workers, and only 2.0% of the Brazilian population was fully vaccinated on the date of censure for this analysis (March 31, 2021) [88].

## 5. Conclusion

The present study is the first to report an association between the NLRP3 rs1539019 polymorphism and CARD8 rs2043211 polymorphisms and protection against disease severity in SARS-CoV-2-infected individuals. We conclude that inflammasome genetic variants influence the COVID-19 clinical outcomes among the patients included in our study. Our work highlights the importance of genetic variations in inflammasome genes in the clinical evolution of COVID-19.

## Figures and Tables

**Table 1:** Sociodemographic and clinical features associated with presentation of either mild and moderate COVID-19 symptoms or severe and critical COVID-19 symptoms (*n* = 433).

Features		WHO scale		aOR^a^ (95% CI)	*P* value^b^	Adjusted *P* value^b^
		Mild and moderate group *N* = 76	Severe and critical group *N* = 357			
Gender; *n* (%)	Female	43 (56.58%)	168 (47.06%)	Reference		
	Male	33 (43.42%)	189 (52.94%)	1.58 (0.82-3.02)	0.169	1
Skin color; *n* (%)	White	36 (47.37%)	64 (17.93%)	Reference		
	Brown	32 (42.11%)	229 (64.15%)	2.76 (1.32-5.77)	0.014	0.562
	Other	8 (10.53%)	64 (17.93%)	2.78 (0.95-8.16)	0.063	1
Age; *n* (%)	40-60	25 (36.76%)	135 (39.59%)	Reference		
	18-40	22 (32.35%)	39 (11.44%)	0.49 (0.13-1.91)	0.914	1
	60-80	18 (26.47%)	139 (40.76%)	1 (0.25-4.06)	1	1
	80-90.6	3 (4.41%)	28 (8.21%)	2.22 (0.11-43.98)	1	1
Schooling; *n* (%)	University education	38 (54.29%)	45 (15.62%)	Reference		
	High school	15 (21.43%)	109 (37.85%)	2.77 (1.19-6.46)	0.036	1
	Low education	17 (24.29%)	134 (46.53%)	2.14 (0.92-4.99)	0.078	1
Diabetes mellitus; *n* (%)	No	66 (86.84%)	235 (65.83%)	Reference		
	Yes	10 (13.16%)	122 (34.17%)	2.01 (0.8-5.05)	0.135	1
Systemic arterial hypertension; *n* (%)	No	59 (77.63%)	177 (49.58%)	Reference		
	Yes	17 (22.37%)	180 (50.42%)	1.57 (0.7-3.51)	0.270	1
Coronary artery disease; *n* (%)	No	67 (88.16%)	347 (97.2%)	Reference		
	Yes	9 (11.84%)	10 (2.8%)	0.21 (0.05-0.89)	0.033	1
Obesity or previous bariatric surgery; *n* (%)	No	69 (90.79%)	291 (81.51%)	Reference		
	Yes	7 (9.21%)	66 (18.49%)	1.14 (0.45-2.87)	0.786	1
O_2_ supplementation or ventilatory support; *n* (%)	No	45 (59.21%)	47 (13.17%)	Reference		
	Yes	31 (40.79%)	310 (86.83%)	5.24 (2.34-11.76)	<0.001	0.002
Saturation below 95%; *n* (%)	No	67 (88.16%)	166 (46.5%)	Reference		
	Yes	9 (11.84%)	191 (53.5%)	8.06 (3.12-20.82)	<0.001	0.001
Fever; *n* (%)	No	42 (55.26%)	149 (41.74%)	Reference		
	Yes	34 (44.74%)	208 (58.26%)	2.05 (1.05-4.01)	0.035	1
Cough; *n* (%)	Yes	45 (59.21%)	225 (63.03%)	Reference		
	No	31 (40.79%)	132 (36.97%)	0.97 (0.49-1.9)	0.925	1
Chest pain; *n* (%)	No	64 (84.21%)	325 (91.04%)	Reference		
	Yes	12 (15.79%)	32 (8.96%)	0.55 (0.22-1.36)	0.195	1
Coryza; *n* (%)	No	59 (77.63%)	327 (91.6%)	Reference		
	Yes	17 (22.37%)	30 (8.4%)	0.8 (0.31-2.03)	0.635	1
Dyspnea; *n* (%)	No	40 (52.63%)	101 (28.29%)	Reference		
	Yes	36 (47.37%)	256 (71.71%)	1.67 (0.84-3.34)	0.143	1
Odynophagia; *n* (%)	No	59 (77.63%)	343 (96.08%)	Reference		
	Yes	17 (22.37%)	14 (3.92%)	0.35 (0.13-0.98)	0.046	1
Anosmia; *n* (%)	No	46 (60.53%)	314 (87.96%)	Reference		
	Yes	30 (39.47%)	43 (12.04%)	0.34 (0.16-0.74)	0.006	0.266
Loss of taste; *n* (%)	No	50 (65.79%)	320 (89.64%)	Reference		
	Yes	26 (34.21%)	37 (10.36%)	0.42 (0.19-0.94)	0.034	1
Diarrhea; *n* (%)	No	61 (80.26%)	326 (91.32%)	Reference		
	Yes	15 (19.74%)	31 (8.68%)	0.3 (0.12-0.73)	0.008	0.343
Abdominal pain; *n* (%)	No	68 (89.47%)	346 (96.92%)	Reference		
	Yes	8 (10.53%)	11 (3.08%)	0.39 (0.11-1.32)	0.129	1
Nausea; *n* (%)	No	65 (85.53%)	338 (94.68%)	Reference		
	Yes	11 (14.47%)	19 (5.32%)	0.47 (0.15-1.46)	0.193	1
Headache; *n* (%)	No	48 (63.16%)	298 (83.47%)	Reference		
	Yes	28 (36.84%)	59 (16.53%)	0.5 (0.24-1.04)	0.063	1
Myalgia; *n* (%)	No	44 (57.89%)	272 (76.19%)	Reference		
	Yes	32 (42.11%)	85 (23.81%)	0.69 (0.34-1.37)	0.288	1
Outcomes; *n* (%)	No hospt	43 (56.58%)	0 (0%)	Reference		
	Hospt	33 (43.42%)	357 (100%)	NC		

^a^Odds ratios were adjusted by skin color, schooling, gender, age, and associated comorbidities, such as diabetes mellitus, coronary artery disease, and obesity or previous bariatric disease. ^b^*P* values were calculated using the unconditional logistic regression model. Associations were considered significant at ^∗^*P* < 0.05. *n*: number of individuals in each group; aOR: adjusted odds ratio; 95% CI: 95% confidence interval; NC: not calculated; Hospt: hospitalized; No hospt: not hospitalized.

**Table 2:** Unconditional logistic multiple regression model of risk and protective genetic factors for disease severity in SARS-CoV-2-infected individuals (*n* = 433).

Genes SNP (rs)	Alleles and genotypes	Mild and moderate group *N* = 76	Severe and critical group *N* = 357	aOR^a^ (95% CI)	*P* value^b^
CARD8 rs2043211	A/A	35 (46.05)	202 (56.58)	Reference	
	A/T	32 (42.11)	130 (36.41)	0.5 (0.25-0.99)	0.046
	T/T	9 (11.84)	25 (7)	0.42 (0.13-1.4)	0.157
	A	102 (67.11)	534 (74.79)	Reference	
	T	50 (32.89)	180 (25.21)	0.93 (0.88-0.99)	0.018
	Noncarrier-A	9 (11.84)	25 (7)	Reference	
	Carrier-A	67 (88.16)	332 (93)	1.76 (0.56-5.54)	0.337
	Noncarrier-T	35 (46.05)	202 (56.58)	Reference	
	Carrier-T	41 (53.95)	155 (43.42)	0.48 (0.25-0.93)	0.029
CARD8 rs6509365	A/A	35 (46.05)	180 (50.42)	Reference	
	A/G	31 (40.79)	142 (39.78)	0.7 (0.35-1.4)	0.314
	G/G	10 (13.16)	35 (9.8)	0.53 (0.18-1.57)	0.253
	A	101 (66.45)	502 (70.31)	Reference	
	G	51 (33.55)	212 (29.69)	0.96 (0.9-1.01)	0.135
	Noncarrier-A	10 (13.16)	35 (9.8)	Reference	
	Carrier-A	66 (86.84)	322 (90.2)	1.6 (0.57-4.47)	0.372
	Noncarrier-G	35 (46.05)	180 (50.42)	Reference	
	Carrier-G	41 (53.95)	177 (49.58)	0.66 (0.35-1.27)	0.217
AIM2 rs2276405	C/C	73 (96.05)	345 (96.64)	Reference	
	C/T	3 (3.95)	12 (3.36)	0.95 (0.19-4.74)	0.951
	C	149 (98.03)	702 (98.32)	Reference	
	T	3 (1.97)	12 (1.68)	1.02 (0.83-1.24)	0.878
	Noncarrier-C	76 (100)	76 (100)	Reference	
	Carrier-C	357 (100)	357 (100)	0.3055	0.306
	Noncarrier-T	73 (96.05)	345 (96.64)	Reference	
	Carrier-T	3 (3.95)	12 (3.36)	0.95 (0.19-4.74)	0.951
IFI16 rs1101996	C/C	38 (50)	166 (46.5)	Reference	
	A/A	9 (11.84)	42 (11.76)	0.62 (0.22-1.78)	0.376
	C/A	29 (38.16)	149 (41.74)	0.72 (0.35-1.46)	0.360
	C	105 (69.08)	481 (67.37)	Reference	
	A	47 (30.92)	233 (32.63)	0.98 (0.92-1.04)	0.445
	Noncarrier-C	9 (11.84)	42 (11.76)	Reference	
	Carrier-C	67 (88.16)	315 (88.24)	1.37 (0.51-3.68)	0.533
	Noncarrier-A	38 (50)	166 (46.5)	Reference	
	Carrier-A	38 (50)	191 (53.5)	0.7 (0.36-1.36)	0.287
CASP1 rs572687	G/G	52 (68.42)	243 (68.07)	Reference	
	A/A	5 (6.58)	14 (3.92)	1.44 (0.26-7.86)	0.675
	G/A	19 (25)	100 (28.01)	1.57 (0.75-3.28)	0.228
	G	123 (80.92)	586 (82.07)	Reference	
	A	29 (19.08)	128 (17.93)	1.03 (0.97-1.11)	0.347
	Noncarrier-G	5 (6.58)	14 (3.92)	Reference	
	Carrier-G	71 (93.42)	343 (96.08)	0.8 (0.15-4.28)	0.796
	Noncarrier-A	52 (68.42)	243 (68.07)	Reference	
	Carrier-A	24 (31.58)	114 (31.93)	1.56 (0.77-3.15)	0.219
IL-1*β* rs1143634^c^	G/G	47 (61.84)	228 (63.87)	Reference	
	A/A	2 (2.63)	9 (2.52)	0.53 (0.08-3.39)	0.503
	G/A	27 (35.53)	119 (33.33)	1.17 (0.58-2.39)	0.660
	G	121 (79.61)	575 (80.53)	Reference	
	A	31 (20.39)	137 (19.19)	1 (0.93-1.07)	0.925
	Noncarrier-G	2 (2.63)	10 (2.8)	Reference	
	Carrier-G	74 (97.37)	347 (97.2)	1.98 (0.31-12.49)	0.467
	Noncarrier-A	47 (61.84)	229 (64.15)	Reference	
	Carrier-A	29 (38.16)	128 (35.85)	1.1 (0.55-2.18)	0.79
NLRP3 rs1539019	C/C	24 (31.58)	146 (40.9)	Reference	
	A/A	17 (22.37)	48 (13.45)	0.36 (0.14-0.92)	0.033
	C/A	35 (46.05)	163 (45.66)	0.48 (0.23-1.03)	0.061
	C	83 (54.61)	455 (63.73)	Reference	
	A	69 (45.39)	259 (36.27)	0.93 (0.88-0.98)	0.010
	Noncarrier-C	17 (22.37)	48 (13.45)	Reference	
	Carrier-C	59 (77.63)	309 (86.55)	1.79 (0.81-3.97)	0.152
	Noncarrier-A	24 (31.58)	146 (40.9)	Reference	
	Carrier-A	52 (68.42)	211 (59.1)	0.45 (0.22-0.91)	0.027
NLRP3 rs4612666	C/C	35 (46.05)	142 (39.78)	Reference	
	C/T	37 (48.68)	155 (43.42)	1.42 (0.72-2.82)	0.316
	T/T	4 (5.26)	60 (16.81)	3.41 (0.93-12.59)	0.065
	C	107 (70.39)	439 (61.48)	Reference	
	T	45 (29.61)	275 (38.52)	1.05 (1-1.11)	0.062
	Noncarrier-C	4 (5.26)	60 (16.81)	Reference	
	Carrier-C	72 (94.74)	297 (83.19)	0.35 (0.1-1.24)	0.103
	Noncarrier-T	35 (46.05)	142 (39.78)	Reference	
	Carrier-T	41 (53.95)	215 (60.22)	1.66 (0.86-3.21)	0.131
NLRP3 rs3806268	G/G	31 (40.79)	135 (37.82)	Reference	
	A/A	10 (13.16)	50 (14.01)	1.34 (0.46-3.94)	0.593
	G/A	35 (46.05)	172 (48.18)	1.4 (0.7-2.79)	0.342
	G	97 (63.82)	442 (61.9)	Reference	
	A	55 (36.18)	272 (38.1)	1.02 (0.97-1.08)	0.476
	Noncarrier-G	10 (13.16)	50 (14.01)	Reference	
	Carrier-G	66 (86.84)	307 (85.99)	0.9 (0.33-2.46)	0.835
	Noncarrier-A	31 (40.79)	135 (37.82)	Reference	
	Carrier-A	45 (59.21)	222 (62.18)	1.39 (0.72-2.68)	0.332
NLRP3 rs35829419	C/C	73 (96.05)	340 (95.24)	Reference	
	A/A	0 (0)	1 (0.28)	NC	
	C/A	3 (3.95)	16 (4.48)	0.73 (0.18-3.02)	0.665
	C	149 (98.03)	696 (97.48)	Reference	
	A	3 (1.97)	18 (2.52)	0.98 (0.84-1.15)	0.827
	Noncarrier-C	0 (0)	1 (0.28)	Reference	
	Carrier-C	76 (100)	356 (99.72)	NC	
	Noncarrier-A	73 (96.05)	340 (95.24)	Reference	
	Carrier-A	3 (3.95)	17 (4.76)	0.75 (0.18-3.08)	0.689
NLRP3 rs10754558	C/C	26 (34.21)	149 (41.74)	Reference	
	C/G	43 (56.58)	164 (45.94)	0.7 (0.35-1.4)	0.313
	G/G	7 (9.21)	44 (12.32)	1.14 (0.38-3.4)	0.819
	C	95 (62.5)	462 (64.71)	Reference	
	G	57 (37.5)	252 (35.29)	0.99 (0.94-1.04)	0.711
	Noncarrier-C	7 (9.21)	44 (12.32)	Reference	
	Carrier-C	69 (90.79)	313 (87.68)	0.71 (0.26-1.96)	0.510
	Noncarrier-G	26 (34.21)	149 (41.74)	Reference	
	Carrier-G	50 (65.79)	208 (58.26)	0.77 (0.39-1.5)	0.435

^a^Odds ratios were adjusted by skin color, schooling, gender, age, and associated comorbidities, such as diabetes mellitus, coronary artery disease, and obesity or previous bariatric disease. ^b^*P* values were calculated using the unconditional logistic regression model. Associations were considered significant at a value of ^∗^*P* < 0.05. ^c^The rs1143634 polymorphism in the IL-1*β* gene determination was not possible for one individual in the hospitalized group. *n*: number of individuals in each group; aOR: adjusted odds ratio; 95% CI: 95% confidence interval; NC: not calculated; A, T, G, and C: each allele count, irrespective of the genotype; Carrier-A: total of genotypes with the A allele; Carrier-T: total of genotypes with the T allele; Carrier-C: total of genotypes with the C allele; Carrier-G: total of genotypes with the G allele; Noncarrier-A: total of genotypes without the A allele; Noncarrier-T: total of genotypes without the T allele; Noncarrier-C: total of genotypes without the C allele; Noncarrier-G: total of genotypes without the G allele.

**Table 3:** Association analyses among NLRP3 and CARD8 inflammasome haplotype frequencies and risk/protection factors for disease severity in SARS-CoV-2-infected individuals.

Genes SNP (rs)	Haplotypes	Mild and moderate group *N* = 76	Severe and critical group *N* = 357	Adjusted model	
				aOR^a^ (95% CI)	*P* value^b^
	CTGCC	26 (17.11)	181 (25.71)	Reference	
	ACACC	10 (6.58)	54 (7.67)	0.78 (0.24-2.56)	0.677
	ACACG	21 (13.82)	112 (15.91)	0.58 (0.25-1.38)	0.220
	ACGCC	4 (2.63)	6 (0.85)	0.11 (0.02-0.69)	0.018
	ACGCG	20 (13.16)	37 (5.26)	0.23 (0.09-0.62)	0.004
NLRP3 rs1539019 rs4612666 rs3806268 rs35829419 rs10754558	ATGAG	2 (1.32)	2 (0.28)	0.1 (0.01-1.62)	0.104
	ATGCC	12 (7.89)	41 (5.82)	0.35 (0.11-1.12)	0.077
	ATGCG	0 (0)	2 (0.28)	NC	
	CCACC	17 (11.18)	77 (10.94)	0.77 (0.28-2.1)	0.605
	CCACG	5 (3.29)	21 (2.98)	0.34 (0.07-1.69)	0.186
	CCGAC	0 (0)	1 (0.14)	NC	
	CCGCC	24 (15.79)	92 (13.07)	0.37 (0.16-0.86)	0.022
	CCGCG	6 (3.95)	35 (4.97)	1.87 (0.32-11.02)	0.487
	CTACC	1 (0.66)	4 (0.57)	0.67 (0.01-76.67)	0.87
	CTACG	1 (0.66)	1 (0.14)	1.28 (0.01-164.14)	0.921
	CTGAC	1 (0.66)	1 (0.14)	0.04 (0-0.96)	0.047
	CTGAG	0 (0)	10 (1.42)	NC	
	CTGCG	2 (1.32)	27 (3.84)	1.96 (0.3-12.75)	0.480
CARD8 rs2043211 rs6509365	AA	99 (65.13)	501 (70.17)	Reference	
	AG	3 (1.97)	33 (4.62)	1.7 (0.43-6.75)	0.449
	TA	2 (1.32)	1 (0.14)	NC	
	TG	48 (31.58)	179 (25.07)	0.61 (0.37-1.02)	0.062

^a^Odds ratios were adjusted by skin color, schooling, gender, age, and associated comorbidities, such as diabetes mellitus, coronary artery disease, and obesity or previous bariatric disease. ^b^*P* values were calculated using the unconditional logistic regression model. Associations were considered significant at a value of ^∗^*P* < 0.05. aOR: adjusted odds ratio; 95% CI: 95% confidence interval; NC: not calculated; *n*: number of individuals in each group.

## Data Availability

The databases used and/or analyzed during the current study would be available from the corresponding author on reasonable request after anonymization.

## Supplementary Materials

Supplementary Materials Table S1: characteristics of inflammasome SNPs included in the study. Table S2: unconditional logistic multiple regression model of risk and protection genetic factors for coronary artery disease in SARS-CoV-2-infected individuals in our cohort (*n* = 433). Table S3: unconditional logistic multiple regression model of risk and protection genetic factors for diabetes mellitus in SARS-CoV-2-infected individuals in our cohort (*n* = 433). Table S4: unconditional logistic multiple regression model of risk and protection genetic factors for obesity or previous bariatric disease in SARS-CoV-2-infected individuals in our cohort (*n* = 433). Table S5: association analyses among NLRP3 and CARD8 inflammasome haplotype frequencies and risk/protection factors for coronary artery disease in SARS-CoV-2-infected individuals.
