# Supplementary material for: Inflammasome Genetic Variants Are Associated with Protection to Clinical Severity of COVID-19 among Patients from Rio de Janeiro, Brazil
Source: Biomed Res Int. 2022 Sep 5;2022:9082455. doi: 10.1155/2022/9082455 (PMC9467712; doi:10.1155/2022/9082455)
Supplement: Supplementary Materials — Table S1: characteristics of inflammasome SNPs included in the study. Table S2: unconditional logistic multiple regression model of risk and protection genetic factors for coronary artery disease in SARS-CoV-2-infected individuals in our cohort (n = 433). Table S3: unconditional logistic multiple regression model of risk and protection genetic factors for diabetes mellitus in SARS-CoV-2-infected individuals in our cohort (n = 433). Table S4: unconditional logistic multiple regression model of risk and protection genetic factors for obesity or previous bariatric disease in SARS-CoV-2-infected individuals in our cohort (n = 433). Table S5: association analyses among NLRP3 and CARD8 inflammasome haplotype frequencies and risk/protection factors for coronary artery disease in SARS-CoV-2-infected individuals. [file 9082455.f1.zip › AJE Editing Certificate.pdf]

Editing Certificate

This document certifies that the manuscript

INFLAMMASOMES GENETIC VARIANTS ARE ASSOCIATED WITH PROTECTION TO CLINICAL SEVERITY OF COVID-19 AMONG PATIENTS FROM RIO DE JANEIRO, BRAZIL

prepared by the authors

Nathalia Beatriz Ramos de Sá, Milena Neira-Goulart, Marcelo Ribeiro-Alves, Hugo Perazzo, Kim Mattos Geraldo, Maria Pia Diniz Ribeiro, Sandra Wagner Cardoso, Beatriz Grinsztejn, Valdiléa G. Veloso, Artur Capão, Marilda Mendonça Siqueira, Ohanna Cavalcanti de Lima Bezerra, Cristiana Couto Garcia, Larissa Rodrigues Gomes, Andressa da Silva Cazote, Dalziza Victalina de Almeida, Carmem Beatriz Wagner Giacoia-Gripp, Fernanda Heloise Côrtes, Mariza Gonçalves Morgado

was edited for proper English language, grammar, punctuation, spelling, and overall style by one or more of the highly qualified native English speaking editors at AJE.

This certificate was issued on **August 11, 2022** and may be verified on the AJE website using the verification code **001A-31ED-335F-6CC6-58D8**.

Neither the research content nor the authors' intentions were altered in any way during the editing process. Documents receiving this certification should be English-ready for publication; however, the author has the ability to accept or reject our suggestions and changes. To verify the final AJE edited version, please visit our verification page at aje.com/certificate. If you have any questions or concerns about this edited document, please contact AJE at support@aje.com.

AJE provides a range of editing, translation, and manuscript services for researchers and publishers around the world. For more information about our company, services, and partner discounts, please visit aje.com.
