# Supplementary material for: Inflammasome Genetic Variants Are Associated with Protection to Clinical Severity of COVID-19 among Patients from Rio de Janeiro, Brazil
Source: Biomed Res Int. 2022 Sep 5;2022:9082455. doi: 10.1155/2022/9082455 (PMC9467712; doi:10.1155/2022/9082455)
Supplement: Supplementary Materials — Table S1: characteristics of inflammasome SNPs included in the study. Table S2: unconditional logistic multiple regression model of risk and protection genetic factors for coronary artery disease in SARS-CoV-2-infected individuals in our cohort (n = 433). Table S3: unconditional logistic multiple regression model of risk and protection genetic factors for diabetes mellitus in SARS-CoV-2-infected individuals in our cohort (n = 433). Table S4: unconditional logistic multiple regression model of risk and protection genetic factors for obesity or previous bariatric disease in SARS-CoV-2-infected individuals in our cohort (n = 433). Table S5: association analyses among NLRP3 and CARD8 inflammasome haplotype frequencies and risk/protection factors for coronary artery disease in SARS-CoV-2-infected individuals. [file 9082455.f1.zip › Supplementary Files.docx]

### Supplementary material

| Genes | SNP ID | Chromosomes | Positions | Localizations | Ancestral allele | Variation allele | *P*-value  HWE^a^ |
| --- | --- | --- | --- | --- | --- | --- | --- |
| CARD8 | rs2043211 | 19 | 48234449 | exonic | A | T | 1 |
| CARD8 | rs6509365 | 19 | 48240212 | intron | A | G | 0.814 |
| AIM2 | rs2276405 | 1 | 159073406 | intron | C | T | 1 |
| IFI16 | rs1101996 | 1 | 159028236 | intron | A | C | 0.814 |
| CASP1 | rs572687 | 11 | 105032992 | intron | A | G | 0.442 |
| IL-1β | rs1143634 | 2 | 112832813 | exonic | G | A | 0.780 |
| NLRP3 | rs3806268 | 1 | 247424175 | exonic | A | G | 1 |
| NLRP3 | rs35829419 | 1 | 247425556 | exonic | A | C | 0.442 |
| NLRP3 | rs4612666 | 1 | 247435768 | intron | C | T | 1 |
| NLRP3 | rs15390193 | 1 | 247436999 | intron | A | C | 1 |
| NLRP3 | rs10754558 | 1 | 247448734 | 3′UTR | C | G | 0.442 |

**Table S1:** Characteristics of inflammasome SNPs included in the study.

SNP: single nucleotide polymorphism; CARD8: Caspase Recruitment Domain-Containing Protein 8; AIM2: Absent In Melanoma 2; IFI16: Interferon Gamma Inducible Protein 16; CASP1: Caspase 1; IL-1β: Interleukin 1 Beta; NLRP3: NLR family Pyrin Domain Containing 3. ^b^P-value of the Hardy- Weinberg equilibrium.

**Table S2:** Unconditional logistic multiple regression model of risk and protection genetic factors for coronary artery disease in SARS-CoV-2 infected individuals in our cohort (n=433).

| Genes  SNP (rs) |  | Alleles and  Genotypes | With Coronary Artery Disease | Without Coronary Artery Disease | aOR^a^  (CI 95%) | | *P*-value^b^ |
| --- | --- | --- | --- | --- | --- | --- | --- |
| CARD8 rs2043211 | | A/A | 261 (54.95) | 9 (47.37) | Reference | | |
|  |  | A/T | 179 (37.68) | 6 (31.58) | 0.73 (0.2-2.72) | | 0.641 |
|  |  | T/T | 35 (7.37) | 4 (21.05) | 5.01 (0.94-26.57) | | 0.058 |
|  |  | A | 701 (73.79) | 24 (63.16) | Reference | | |
|  |  | T | 249 (26.21) | 14 (36.84) | 1.01 (0.98-1.05) | | 0.426 |
|  |  | NonCarrier-A | 35 (7.37) | 4 (21.05) | Reference | | |
|  |  | **Carrier-A** | 440 (92.63) | 15 (78.95) | 0.17 (0.04-0.85) | | **0.031** |
|  |  | NonCarrier-T | 261 (54.95) | 9 (47.37) | Reference | | |
|  |  | Carrier-T | 214 (45.05) | 10 (52.63) | 1.14 (0.37-3.53) | | 0.816 |
| CARD8  rs6509365 | | A/A | 236 (49.68) | 8 (42.11) | Reference | | |
|  |  | A/G | 194 (40.84) | 6 (31.58) | 0.83 (0.23-2.98) | | 0.777 |
|  |  | G/G | 45 (9.47) | 5 (26.32) | 3.16 (0.65-15.31) | | 0.152 |
|  |  | A | 666 (70.11) | 22 (57.89) | Reference | | |
|  |  | G | 284 (29.89) | 16 (42.11) | 1.01 (0.98-1.04) | | 0.466 |
|  |  | NonCarrier-A | 45 (9.47) | 5 (26.32) | Reference | | |
|  |  | Carrier-A | 430 (90.53) | 14 (73.68) | 0.29 (0.07-1.31) | | 0.108 |
|  |  | NonCarrier-G | 236 (49.68) | 8 (42.11) | Reference | | |
|  |  | Carrier-G | 239 (50.32) | 11 (57.89) | 1.2 (0.39-3.69) | | 0.757 |
| AIM2  rs2276405 | | C/C | 457 (96.21) | 18 (94.74) | Reference | | |
|  |  | C/T | 18 (3.79) | 1 (5.26) | 1.05 (0.1-11.16) | | 0.968 |
|  |  | C | 932 (98.11) | 37 (97.37) | Reference | | |
|  |  | T | 18 (1.89) | 1 (2.63) | 1.02 (0.93-1.13) | | 0.653 |
|  |  | NonCarrier-C | 475 (100) | 475 (100) | Reference | | |
|  |  | Carrier-C | 19 (100) | 19 (100) | 0 | | 0 |
|  |  | NonCarrier-T | 457 (96.21) | 18 (94.74) | Reference | | |
|  |  | Carrier-T | 18 (3.79) | 1 (5.26) | 1.05 (0.1-11.16) | | 0.968 |
| IFI16  rs1101996 | | C/C | 222 (46.74) | 8 (42.11) | Reference | | |
|  |  | A/A | 58 (12.21) | 3 (15.79) | 1.05 (0.19-5.84) | | 0.956 |
|  |  | C/A | 195 (41.05) | 8 (42.11) | 1.23 (0.37-4.09) | | 0.734 |
|  |  | C | 639 (67.26) | 24 (63.16) | Reference | | |
|  |  | A | 311 (32.74) | 14 (36.84) | 1.01 (0.98-1.04) | | 0.681 |
|  |  | NonCarrier-C | 58 (12.21) | 3 (15.79) | Reference | | |
|  |  | Carrier-C | 417 (87.79) | 16 (84.21) | 1.04 (0.21-5.3) | | 0.958 |
|  |  | NonCarrier-A | 222 (46.74) | 8 (42.11) | Reference | | |
|  |  | Carrier-A | 253 (53.26) | 11 (57.89) | 1.18 (0.39-3.6) | | 0.771 |
| CASP1  rs572687 | | G/G | 324 (68.21) | 12 (63.16) | Reference | | |
|  |  | A/A | 20 (4.21) | 1 (5.26) | 3.66 (0.35-38.44) | | 0.279 |
|  |  | G/A | 131 (27.58) | 6 (31.58) | 1.27 (0.38-4.25) | | 0.698 |
|  |  | G | 779 (82) | 30 (78.95) | Reference | | |
|  |  | A | 171 (18) | 8 (21.05) | 1.01 (0.97-1.05) | | 0.608 |
|  |  | NonCarrier-G | 20 (4.21) | 1 (5.26) | Reference | | |
|  |  | Carrier-G | 455 (95.79) | 18 (94.74) | 0.3 (0.03-2.98) | | 0.303 |
|  |  | NonCarrier-A | 324 (68.21) | 12 (63.16) | Reference | | |
|  |  | Carrier-A | 151 (31.79) | 7 (36.84) | 1.44 (0.46-4.51) | | 0.536 |
| IL-1β  rs1143634^c^ | | G/G | 302 (63.71) | 12 (63.16) | Reference | | |
|  |  | A/A | 13 (2.74) | 0 (0) | 0 (0-Inf) | | 0.996 |
|  |  | G/A | 159 (33.54) | 7 (36.84) | 0.93 (0.3-2.95) | | 0.908 |
|  |  | G | 763 (80.49) | 31 (81.58) | Reference | | |
|  |  | A | 185 (19.51) | 7 (18.42) | 0.99 (0.96-1.03) | | 0.718 |
|  |  | NonCarrier-G | 13 (2.74) | 0 (0) | Reference | | |
|  |  | Carrier-G | 461 (97.26) | 19 (100) | 8952448.82 (0-Inf) | | 0.996 |
|  |  | NonCarrier-A | 302 (63.71) | 12 (63.16) | Reference | | |
|  |  | Carrier-A | 172 (36.29) | 7 (36.84) | 0.88 (0.28-2.78) | | 0.829 |
| NLRP3  rs1539019 | | C/C | 195 (41.05) | 9 (47.37) | Reference | | |
|  |  | A/A | 70 (14.74) | 2 (10.53) | 0.28 (0.03-2.48) | | 0.251 |
|  |  | C/A | 210 (44.21) | 8 (42.11) | 0.83 (0.26-2.66) | | 0.750 |
|  |  | C | 600 (63.16) | 26 (68.42) | Reference | | |
|  |  | A | 350 (36.84) | 12 (31.58) | 0.98 (0.95-1.01) | | 0.224 |
|  |  | NonCarrier-C | 70 (14.74) | 2 (10.53) | Reference | | |
|  |  | Carrier-C | 405 (85.26) | 17 (89.47) | 3.26 (0.4-26.71) | | 0.272 |
|  |  | NonCarrier-A | 195 (41.05) | 9 (47.37) | Reference | | |
|  |  | Carrier-A | 280 (58.95) | 10 (52.63) | 0.66 (0.21-2.02) | | 0.467 |
| NLRP3  rs4612666 | | C/C | 200 (42.11) | 4 (21.05) | Reference | | |
|  |  | C/T | 203 (42.74) | 11 (57.89) | 3.74 (0.93-14.99) | | 0.063 |
|  |  | T/T | 72 (15.16) | 4 (21.05) | 2.53 (0.35-18.36) | | 0.359 |
|  |  | C | 603 (63.47) | 19 (50) | Reference | | |
|  |  | T | 347 (36.53) | 19 (50) | 1.02 (0.99-1.05) | | 0.150 |
|  |  | NonCarrier-C | 72 (15.16) | 4 (21.05) | Reference | | |
|  |  | Carrier-C | 403 (84.84) | 15 (78.95) | 0.92 (0.17-4.96) | | 0.919 |
|  |  | NonCarrier-T | 200 (42.11) | 4 (21.05) | Reference | | |
|  |  | Carrier-T | 275 (57.89) | 15 (78.95) | 3.48 (0.89-13.56) | | 0.072 |
| NLRP3  rs3806268 | | G/G | 185 (38.95) | 8 (42.11) | Reference | | |
|  |  | A/A | 69 (14.53) | 2 (10.53) | 0.94 (0.16-5.53) | | 0.950 |
|  |  | G/A | 221 (46.53) | 9 (47.37) | 0.73 (0.22-2.42) | | 0.612 |
|  |  | G | 591 (62.21) | 25 (65.79) | Reference | | |
|  |  | A | 359 (37.79) | 13 (34.21) | 1 (0.97-1.02) | | 0.770 |
|  |  | NonCarrier-G | 69 (14.53) | 2 (10.53) | Reference | | |
|  |  | Carrier-G | 406 (85.47) | 17 (89.47) | 0.88 (0.17-4.5) | | 0.880 |
|  |  | NonCarrier-A | 185 (38.95) | 8 (42.11) | Reference | | |
|  |  | Carrier-A | 290 (61.05) | 11 (57.89) | 0.77 (0.25-2.41) | | 0.655 |
| NLRP3  rs35829419 | | C/C | 457 (96.21) | 17 (89.47) | Reference | | |
|  |  | A/A | 1 (0.21) | 0 (0) | 0.67 (0-Inf) | 1 | |
|  |  | C/A | 17 (3.58) | 2 (10.53) | 3.32 (0.31-35.17) | | 0.319 |
|  |  | C | 931 (98) | 36 (94.74) | Reference | | |
|  |  | A | 19 (2) | 2 (5.26) | 1.02 (0.93-1.12) | | 0.649 |
|  |  | NonCarrier-C | 1 (0.21) | 0 (0) | Reference | | |
|  |  | Carrier-C | 474 (99.79) | 19 (100) | 2.16 (0-Inf) | 1 | |
|  |  | NonCarrier-A | 457 (96.21) | 17 (89.47) | Reference | | |
|  |  | Carrier-A | 18 (3.79) | 2 (10.53) | 3.32 (0.31-35.17) | | 0.319 |
| NLRP3  rs10754558 | | C/C | 202 (42.53) | 5 (26.32) | Reference | | |
|  |  | C/G | 218 (45.89) | 10 (52.63) | 1.98 (0.48-8.13) | | 0.345 |
|  |  | G/G | 55 (11.58) | 4 (21.05) | 4.29 (0.83-22.15) | | 0.082 |
|  |  | C | 95 (62.5) | 462 (64.71) | Reference | | |
|  |  | G | 57 (37.5) | 252 (35.29) | 1.02 (1-1.05) | | 0.097 |
|  |  | NonCarrier-C | 7 (9.21) | 44 (12.32) | Reference | | |
|  |  | Carrier-C | 69 (90.79) | 313 (87.68) | 0.37 (0.1-1.32) | | 0.125 |
|  |  | NonCarrier-G | 26 (34.21) | 149 (41.74) | Reference | | |
|  |  | Carrier-G | 50 (65.79) | 208 (58.26) | 2.41 (0.62-9.29) | | 0.202 |

^a^Odds ratios were adjusted by skin color, schooling, gender, age, and associated comorbidities such as diabetes mellitus, and obesity or previous bariatric disease. ^b^*P*-values were calculated using the unconditional logistic regression model. Associations were considered significant with a value of * *P* < 0.05. ^c^The rs1143634 polymorphism in the IL-1β gene determination was not possible for one individual from the hospitalized group. N: number of individuals in each group; aOR: adjusted odds ratio; 95% CI: 95% confidence interval; A, T, G, and C = each allele count, irrespective of the genotype. Carrier-A = total of genotypes with the A allele; Carrier-T = total of genotypes with T allele; Carrier-C = total of genotypes with the C allele; Carrier-G = total of genotypes with the G allele; Non-Carrier-A = total of genotypes without the A allele; Non-Carrier-T = total of genotypes without the T allele; Non-Carrier-C = total of genotypes without the C allele; Non-Carrier-G = total of genotypes without the G allele.

**Table S3:** Unconditional logistic multiple regression model of risk and protection genetic factors for diabetes mellitus in SARS-CoV-2 infected individuals in our cohort (n=433).

| Genes  SNP (rs) |  | Alleles and  Genotypes | With Diabetes Mellitus | Without Diabetes Mellitus | aOR^a^  (CI 95%) | | *P*-value^b^ | |
| --- | --- | --- | --- | --- | --- | --- | --- | --- |
| CARD8 rs2043211 | | A/A | 197 (55.65) | 73 (52.14) | Reference | | | |
|  |  | A/T | 132 (37.29) | 53 (37.86) | 1.27 (0.75-2.16) | | 0.368 | |
|  |  | T/T | 25 (7.06) | 14 (10) | 1.43 (0.54-3.75) | | 0.468 | |
|  |  | A | 526 (74.29) | 199 (71.07) | Reference | | | |
|  |  | T | 182 (25.71) | 81 (28.93) | 1.04 (0.97-1.11) | | 0.330 | |
|  |  | NonCarrier-A | 25 (7.06) | 14 (10) | Reference | | | |
|  |  | Carrier-A | 329 (92.94) | 126 (90) | 0.78 (0.3-1.98) | | 0.595 | |
|  |  | NonCarrier-T | 197 (55.65) | 73 (52.14) | Reference | | | |
|  |  | Carrier-T | 157 (44.35) | 67 (47.86) | 1.3 (0.79-2.14) | | 0.309 | |
| CARD8  rs6509365 | | A/A | 178 (50.28) | 66 (47.14) | Reference | | | |
|  |  | A/G | 141 (39.83) | 59 (42.14) | 1.39 (0.82-2.36) | | 0.225 | |
|  |  | G/G | 35 (9.89) | 15 (10.71) | 0.93 (0.38-2.24) | | 0.866 | |
|  |  | A | 497 (70.2) | 191 (68.21) | Reference | | | |
|  |  | G | 211 (29.8) | 89 (31.79) | 1.01 (0.95-1.08) | | 0.679 | |
|  |  | NonCarrier-A | 35 (9.89) | 15 (10.71) | Reference | | | |
|  |  | Carrier-A | 319 (90.11) | 125 (89.29) | 1.26 (0.54-2.93) | | 0.591 | |
|  |  | NonCarrier-G | 178 (50.28) | 66 (47.14) | Reference | | | |
|  |  | Carrier-G | 176 (49.72) | 74 (52.86) | 1.28 (0.78-2.12) | | 0.331 | |
| AIM2  rs2276405 | | C/C | 338 (95.48) | 137 (97.86) | Reference | | | |
|  |  | C/T | 16 (4.52) | 3 (2.14) | 0.43 (0.09-2.16) | | 0.306 | |
|  |  | C | 692 (97.74) | 277 (98.93) | Reference | | | |
|  |  | T | 16 (2.26) | 3 (1.07) | 0.9 (0.73-1.12) | | 0.354 | |
|  |  | NonCarrier-C | 354 (100) | 354 (100) | Reference | | | |
|  |  | Carrier-C | 140 (100) | 140 (100) | 0 | | 0 | |
|  |  | NonCarrier-T | 338 (95.48) | 137 (97.86) | Reference | | | |
|  |  | Carrier-T | 16 (4.52) | 3 (2.14) | 0.43 (0.09-2.16) | | 0.306 | |
| IFI16  rs1101996 | | C/C | 177 (50) | 53 (37.86) | Reference | | | |
|  |  | A/A | 41 (11.58) | 20 (14.29) | 1.46 (0.63-3.37) | | 0.373 | |
|  |  | C/A | 136 (38.42) | 67 (47.86) | 1.29 (0.76-2.19) | | 0.353 | |
|  |  | C | 490 (69.21) | 173 (61.79) | Reference | | | |
|  |  | A | 218 (30.79) | 107 (38.21) | 1.04 (0.97-1.11) | | 0.271 | |
|  |  | NonCarrier-C | 41 (11.58) | 20 (14.29) | Reference | | | |
|  |  | Carrier-C | 313 (88.42) | 120 (85.71) | 0.78 (0.36-1.72) | | 0.540 | |
|  |  | NonCarrier-A | 177 (50) | 53 (37.86) | Reference | | | |
|  |  | Carrier-A | 177 (50) | 87 (62.14) | 1.32 (0.79-2.19) | | 0.286 | |
| CASP1  rs572687 | | G/G | 246 (69.49) | 90 (64.29) | Reference | | | |
|  |  | A/A | 16 (4.52) | 5 (3.57) | 1.47 (0.35-6.18) | | 0.599 | |
|  |  | G/A | 92 (25.99) | 45 (32.14) | 1.53 (0.89-2.61) | | 0.122 | |
|  |  | G | 584 (82.49) | 225 (80.36) | Reference | | | |
|  |  | A | 124 (17.51) | 55 (19.64) | 1.06 (0.98-1.15) | | 0.155 | |
|  |  | NonCarrier-G | 16 (4.52) | 5 (3.57) | Reference | | | |
|  |  | Carrier-G | 338 (95.48) | 135 (96.43) | 0.78 (0.19-3.22) | | 0.727 | |
|  |  | NonCarrier-A | 246 (69.49) | 90 (64.29) | Reference | | | |
|  |  | Carrier-A | 108 (30.51) | 50 (35.71) | 1.52 (0.9-2.56) | | 0.114 | |
| IL-1β  rs1143634^c^ | | G/G | 225 (63.74) | 89 (63.57) | Reference | | | |
|  |  | A/A | 10 (2.83) | 3 (2.14) | 0.65 (0.15-2.8) | | 0.567 | |
|  |  | G/A | 118 (33.43) | 48 (34.29) | 1.08 (0.64-1.84) | | 0.766 | |
|  |  | G | 568 (80.45) | 226 (80.71) | Reference | | | |
|  |  | A | 138 (19.55) | 54 (19.29) | 1 (0.92-1.08) | | 0.953 | |
|  |  | NonCarrier-G | 10 (2.83) | 3 (2.14) | Reference | | | |
|  |  | Carrier-G | 343 (97.17) | 137 (97.86) | 1.57 (0.37-6.62) | | 0.542 | |
|  |  | NonCarrier-A | 225 (63.74) | 89 (63.57) | Reference | | | |
|  |  | Carrier-A | 128 (36.26) | 51 (36.43) | 1.03 (0.62-1.72) | | 0.898 | |
| NLRP3  rs1539019 | | C/C | 146 (41.24) | 58 (41.43) | Reference | | | |
|  |  | A/A | 48 (13.56) | 24 (17.14) | 1.99 (0.98-4.06) | | 0.057 | |
|  |  | C/A | 160 (45.2) | 58 (41.43) | 0.88 (0.51-1.52) | | 0.645 | |
|  |  | C | 452 (63.84) | 174 (62.14) | Reference | | | |
|  |  | A | 256 (36.16) | 106 (37.86) | 1.05 (0.98-1.12) | | 0.138 | |
|  |  | NonCarrier-C | 48 (13.56) | 24 (17.14) | Reference | | | |
|  |  | **Carrier-C** | 306 (86.44) | 116 (82.86) | 0.47 (0.24-0.91) | | **0.024** | |
|  |  | NonCarrier-A | 146 (41.24) | 58 (41.43) | Reference | | | |
|  |  | Carrier-A | 208 (58.76) | 82 (58.57) | 1.11 (0.67-1.83) | | 0.692 | |
| NLRP3  rs4612666 | | C/C | 137 (38.7) | 67 (47.86) | Reference | | | |
|  |  | C/T | 166 (46.89) | 48 (34.29) | 0.64 (0.37-1.11) | | 0.110 | |
|  |  | T/T | 51 (14.41) | 25 (17.86) | 0.72 (0.35-1.5) | | 0.387 | |
|  |  | C | 440 (62.15) | 182 (65) | Reference | | | |
|  |  | T | 268 (37.85) | 98 (35) | 0.96 (0.9-1.02) | | 0.173 | |
|  |  | NonCarrier-C | 51 (14.41) | 25 (17.86) | Reference | | | |
|  |  | Carrier-C | 303 (85.59) | 115 (82.14) | 1.12 (0.56-2.23) | | 0.741 | |
|  |  | NonCarrier-T | 137 (38.7) | 67 (47.86) | Reference | | | |
|  |  | Carrier-T | 217 (61.3) | 73 (52.14) | 0.66 (0.4-1.1) | | 0.109 | |
| NLRP3  rs3806268 | | G/G | 140 (39.55) | 53 (37.86) | Reference | | | |
|  |  | A/A | 47 (13.28) | 24 (17.14) | 1.76 (0.81-3.84) | | 0.153 | |
|  |  | G/A | 167 (47.18) | 63 (45) | 1.19 (0.69-2.07) | | 0.528 | |
|  |  | G | 447 (63.14) | 169 (60.36) | Reference | | | |
|  |  | A | 261 (36.86) | 111 (39.64) | 1.04 (0.98-1.11) | | 0.190 | |
|  |  | NonCarrier-G | 47 (13.28) | 24 (17.14) | Reference | | | |
|  |  | Carrier-G | 307 (86.72) | 116 (82.86) | 0.63 (0.31-1.28) | | 0.198 | |
|  |  | NonCarrier-A | 140 (39.55) | 53 (37.86) | Reference | | | |
|  |  | Carrier-A | 214 (60.45) | 87 (62.14) | 1.3 (0.77-2.2) | | 0.324 | |
| NLRP3  rs35829419 | | C/C | 344 (97.18) | 130 (92.86) | Reference | | | |
|  |  | A/A | 0 (0) | 1 (0.71) | 1840875.47 (0-Inf) | | | 0.987 |
|  |  | C/A | 10 (2.82) | 9 (6.43) | 2.17 (0.74-6.34) | | 0.158 | |
|  |  | C | 698 (98.59) | 269 (96.07) | Reference | | | |
|  |  | A | 10 (1.41) | 11 (3.93) | 1.19 (0.98-1.44) | | 0.078 | |
|  |  | NonCarrier-C | 0 (0) | 1 (0.71) | Reference | | | |
|  |  | Carrier-C | 354 (100) | 139 (99.29) | 0 (0-Inf) | 0.987 | | |
|  |  | NonCarrier-A | 344 (97.18) | 130 (92.86) | Reference | | | |
|  |  | Carrier-A | 10 (2.82) | 10 (7.14) | 2.33 (0.82-6.63) | | 0.111 | |
| NLRP3  rs10754558 | | C/C | 151 (42.66) | 56 (40) | Reference | | | |
|  |  | C/G | 161 (45.48) | 67 (47.86) | 1.29 (0.75-2.21) | | 0.360 | |
|  |  | G/G | 42 (11.86) | 17 (12.14) | 1.45 (0.65-3.25) | | 0.362 | |
|  |  | C | 463 (65.4) | 179 (63.93) | Reference | | | |
|  |  | G | 245 (34.6) | 101 (36.07) | 1.04 (0.97-1.1) | | 0.282 | |
|  |  | NonCarrier-C | 42 (11.86) | 17 (12.14) | Reference | | | |
|  |  | Carrier-C | 312 (88.14) | 123 (87.86) | 0.8 (0.38-1.66) | | 0.544 | |
|  |  | NonCarrier-G | 151 (42.66) | 56 (40) | Reference | | | |
|  |  | Carrier-G | 203 (57.34) | 84 (60) | 1.32 (0.79-2.21) | | 0.294 | |

^a^Odds ratios were adjusted by skin color, schooling, gender, age, and associated comorbidities such as coronary artery disease and obesity or previous bariatric disease. ^b^*P*-values were calculated using the unconditional logistic regression model. Associations were considered significant with a value of * *P* < 0.05. ^c^The rs1143634 polymorphism in the IL-1β gene determination was not possible for one individual from the hospitalized group. N: number of individuals in each group; aOR: adjusted odds ratio; 95% CI: 95% confidence interval; A, T, G, and C = each allele count, irrespective of the genotype. Carrier-A = total of genotypes with the A allele; Carrier-T = total of genotypes with T allele; Carrier-C = total of genotypes with the C allele; Carrier-G = total of genotypes with the G allele; Non-Carrier-A = total of genotypes without the A allele; Non-Carrier-T = total of genotypes without the T allele; Non-Carrier-C = total of genotypes without the C allele; Non-Carrier-G = total of genotypes without the G allele.

**Table S4:** Unconditional logistic multiple regression model of risk and protection genetic factors for obesity or previous bariatric disease in SARS-CoV-2 infected individuals in our cohort (n=433).

| Genes  SNP (rs) |  | Alleles and  Genotypes | With obesity or previous bariatric disease | Without obesity or previous bariatric disease | aOR^a^  (CI 95%) | | *P*-value^b^ | |
| --- | --- | --- | --- | --- | --- | --- | --- | --- |
| CARD8 rs2043211 | | A/A | 224 (54.37) | 46 (56.1) | Reference | | | |
|  |  | A/T | 155 (37.62) | 30 (36.59) | 0.88 (0.48-1.58) | | 0.659 | |
|  |  | T/T | 33 (8.01) | 6 (7.32) | 0.74 (0.23-2.4) | | 0.618 | |
|  |  | A | 526 (74.29) | 199 (71.07) | Reference | | | |
|  |  | T | 603 (73.18) | 122 (74.39) | 0.98 (0.92-1.04) | | 0.496 | |
|  |  | NonCarrier-A | 221 (26.82) | 42 (25.61) | Reference | | | |
|  |  | Carrier-A | 33 (8.01) | 6 (7.32) | 1.28 (0.4-4.04) | | 0.677 | |
|  |  | NonCarrier-T | 379 (91.99) | 76 (92.68) | Reference | | | |
|  |  | Carrier-T | 224 (54.37) | 46 (56.1) | 0.85 (0.48-1.5) | | 0.583 | |
| CARD8  rs6509365 | | A/A | 202 (49.03) | 42 (51.22) | Reference | | | |
|  |  | A/G | 170 (41.26) | 30 (36.59) | 0.75 (0.41-1.38) | | 0.362 | |
|  |  | G/G | 40 (9.71) | 10 (12.2) | 1.12 (0.45-2.8) | | 0.801 | |
|  |  | A | 574 (69.66) | 114 (69.51) | Reference | | | |
|  |  | G | 250 (30.34) | 50 (30.49) | 0.99 (0.93-1.05) | | 0.747 | |
|  |  | NonCarrier-A | 40 (9.71) | 10 (12.2) | Reference | | | |
|  |  | Carrier-A | 372 (90.29) | 72 (87.8) | 0.78 (0.33-1.88) | | 0.587 | |
|  |  | NonCarrier-G | 202 (49.03) | 42 (51.22) | Reference | | | |
|  |  | Carrier-G | 210 (50.97) | 40 (48.78) | 0.82 (0.47-1.44) | | 0.498 | |
| AIM2  rs2276405 | | C/C | 396 (96.12) | 79 (96.34) | Reference | | | |
|  |  | C/T | 16 (3.88) | 3 (3.66) | 1.23 (0.31-4.87) | | 0.767 | |
|  |  | C | 808 (98.06) | 161 (98.17) | Reference | | | |
|  |  | T | 16 (1.94) | 3 (1.83) | 1.03 (0.85-1.25) | | 0.740 | |
|  |  | NonCarrier-C | 412 (100) | 412 (100) | Reference | | | |
|  |  | Carrier-C | 82 (100) | 82 (100) | 0.1513 | | 0.151 | |
|  |  | NonCarrier-T | 396 (96.12) | 79 (96.34) | Reference | | | |
|  |  | Carrier-T | 16 (3.88) | 3 (3.66) | 1.23 (0.31-4.87) | | 0.767 | |
| IFI16  rs1101996 | | C/C | 191 (46.36) | 39 (47.56) | Reference | | | |
|  |  | A/A | 50 (12.14) | 11 (13.41) | 0.88 (0.33-2.37) | | 0.799 | |
|  |  | C/A | 171 (41.5) | 32 (39.02) | 0.92 (0.51-1.67) | | 0.794 | |
|  |  | C | 553 (67.11) | 110 (67.07) | Reference | | | |
|  |  | A | 271 (32.89) | 54 (32.93) | 0.99 (0.93-1.05) | | 0.732 | |
|  |  | NonCarrier-C | 50 (12.14) | 11 (13.41) | Reference | | | |
|  |  | Carrier-C | 362 (87.86) | 71 (86.59) | 1.09 (0.43-2.79) | | 0.857 | |
|  |  | NonCarrier-A | 191 (46.36) | 39 (47.56) | Reference | | | |
|  |  | Carrier-A | 221 (53.64) | 43 (52.44) | 0.92 (0.52-1.62) | | 0.763 | |
| CASP1  rs572687 | | G/G | 275 (66.75) | 61 (74.39) | Reference | | | |
|  |  | A/A | 18 (4.37) | 3 (3.66) | 1.02 (0.2-5.17) | | 0.984 | |
|  |  | G/A | 119 (28.88) | 18 (21.95) | 0.77 (0.4-1.46) | | 0.416 | |
|  |  | G | 669 (81.19) | 140 (85.37) | Reference | | | |
|  |  | A | 155 (18.81) | 24 (14.63) | 0.98 (0.91-1.05) | | 0.554 | |
|  |  | NonCarrier-G | 18 (4.37) | 3 (3.66) | Reference | | | |
|  |  | Carrier-G | 394 (95.63) | 79 (96.34) | 0.92 (0.18-4.64) | | 0.918 | |
|  |  | NonCarrier-A | 275 (66.75) | 61 (74.39) | Reference | | | |
|  |  | Carrier-A | 137 (33.25) | 21 (25.61) | 0.79 (0.43-1.46) | | 0.449 | |
| IL-1β  rs1143634^c^ | | G/G | 258 (62.77) | 56 (68.29) | Reference | | | |
|  |  | A/A | 9 (2.19) | 4 (4.88) | 1.14 (0.27-4.88) | | 0.857 | |
|  |  | G/A | 144 (35.04) | 22 (26.83) | 0.78 (0.42-1.44) | | 0.427 | |
|  |  | G | 660 (80.29) | 134 (81.71) | Reference | | | |
|  |  | A | 162 (19.71) | 30 (18.29) | 0.98 (0.92-1.05) | | 0.589 | |
|  |  | NonCarrier-G | 9 (2.19) | 4 (4.88) | Reference | | | |
|  |  | Carrier-G | 402 (97.81) | 78 (95.12) | 0.81 (0.19-3.45) | | 0.780 | |
|  |  | NonCarrier-A | 258 (62.77) | 56 (68.29) | Reference | | | |
|  |  | Carrier-A | 153 (37.23) | 26 (31.71) | 0.81 (0.45-1.47) | | 0.492 | |
| NLRP3  rs1539019 | | C/C | 164 (39.81) | 40 (48.78) | Reference | | | |
|  |  | A/A | 63 (15.29) | 9 (10.98) | 0.54 (0.22-1.31) | | 0.174 | |
|  |  | C/A | 185 (44.9) | 33 (40.24) | 0.71 (0.39-1.28) | | 0.256 | |
|  |  | C | 513 (62.26) | 113 (68.9) | Reference | | | |
|  |  | A | 311 (37.74) | 51 (31.1) | 0.95 (0.9-1.01) | | 0.102 | |
|  |  | NonCarrier-C | 63 (15.29) | 9 (10.98) | Reference | | | |
|  |  | Carrier-C | 349 (84.71) | 73 (89.02) | 1.56 (0.68-3.63) | | 0.297 | |
|  |  | NonCarrier-A | 164 (39.81) | 40 (48.78) | Reference | | | |
|  |  | Carrier-A | 248 (60.19) | 42 (51.22) | 0.66 (0.38-1.15) | | 0.146 | |
| NLRP3  rs4612666 | | C/C | 163 (39.56) | 41 (50) | Reference | | | |
|  |  | C/T | 187 (45.39) | 27 (32.93) | 0.93 (0.5-1.74) | | 0.829 | |
|  |  | T/T | 62 (15.05) | 14 (17.07) | 1.37 (0.63-2.98) | | 0.431 | |
|  |  | C | 513 (62.26) | 109 (66.46) | Reference | | | |
|  |  | T | 311 (37.74) | 55 (33.54) | 1.02 (0.96-1.08) | | 0.570 | |
|  |  | NonCarrier-C | 62 (15.05) | 14 (17.07) | Reference | | | |
|  |  | Carrier-C | 350 (84.95) | 68 (82.93) | 0.71 (0.34-1.46) | | 0.350 | |
|  |  | NonCarrier-T | 163 (39.56) | 41 (50) | Reference | | | |
|  |  | Carrier-T | 249 (60.44) | 41 (50) | 1.05 (0.6-1.85) | | 0.866 | |
| NLRP3  rs3806268 | | G/G | 156 (37.86) | 37 (45.12) | Reference | | | |
|  |  | A/A | 57 (13.83) | 14 (17.07) | 0.73 (0.31-1.72) | | 0.474 | |
|  |  | **G/A** | 199 (48.3) | 31 (37.8) | 0.42 (0.23-0.78) | | **0.006** | |
|  |  | G | 511 (62.01) | 105 (64.02) | Reference | | | |
|  |  | A | 313 (37.99) | 59 (35.98) | 0.96 (0.91-1.01) | | 0.130 | |
|  |  | NonCarrier-G | 57 (13.83) | 14 (17.07) | Reference | | | |
|  |  | Carrier-G | 355 (86.17) | 68 (82.93) | 0.86 (0.39-1.91) | | 0.716 | |
|  |  | NonCarrier-A | 156 (37.86) | 37 (45.12) | Reference | | | |
|  |  | **Carrier-A** | 256 (62.14) | 45 (54.88) | 0.48 (0.27-0.85) | | **0.012** | |
| NLRP3  rs35829419 | | C/C | 398 (96.6) | 76 (92.68) | Reference | | | |
|  |  | A/A | 0 (0) | 1 (1.22) | 136985966.82 (0-Inf) | | | 0.996 |
|  |  | C/A | 14 (3.4) | 5 (6.1) | 2.3 (0.73-7.27) | | 0.156 | |
|  |  | C | 810 (98.3) | 157 (95.73) | Reference | | | |
|  |  | **A** | 14 (1.7) | 7 (4.27) | 1.21 (1.02-1.44) | | **0.029** | |
|  |  | NonCarrier-C | 0 (0) | 1 (1.22) | Reference | | | |
|  |  | Carrier-C | 412 (100) | 81 (98.78) | 0 (0-Inf) | 0.996 | | |
|  |  | NonCarrier-A | 398 (96.6) | 76 (92.68) | Reference | | | |
|  |  | Carrier-A | 14 (3.4) | 6 (7.32) | 2.72 (0.91-8.11) | | 0.073 | |
| NLRP3  rs10754558 | | C/C | 168 (40.78) | 39 (47.56) | Reference | | | |
|  |  | C/G | 192 (46.6) | 36 (43.9) | 0.76 (0.43-1.37) | | 0.369 | |
|  |  | G/G | 52 (12.62) | 7 (8.54) | 0.54 (0.2-1.44) | | 0.216 | |
|  |  | C | 528 (64.08) | 114 (69.51) | Reference | | | |
|  |  | G | 296 (35.92) | 50 (30.49) | 0.96 (0.91-1.02) | | 0.158 | |
|  |  | NonCarrier-C | 52 (12.62) | 7 (8.54) | Reference | | | |
|  |  | Carrier-C | 360 (87.38) | 75 (91.46) | 1.62 (0.63-4.15) | | 0.314 | |
|  |  | NonCarrier-G | 168 (40.78) | 39 (47.56) | Reference | | | |
|  |  | Carrier-G | 244 (59.22) | 43 (52.44) | 0.71 (0.41-1.25) | | 0.237 | |

^a^Odds ratios were adjusted by skin color, schooling, gender, age, and associated comorbidities such as coronary artery disease and diabetes mellitus. ^b^*P*-values were calculated using the unconditional logistic regression model. Associations were considered significant with a value of * *P* < 0.05. ^c^The rs1143634 polymorphism in the IL-1β gene determination was not possible for one individual from the hospitalized group. N: number of individuals in each group; aOR: adjusted odds ratio; 95% CI: 95% confidence interval; A, T, G, and C = each allele count, irrespective of the genotype. Carrier-A = total of genotypes with the A allele; Carrier-T = total of genotypes with T allele; Carrier-C = total of genotypes with the C allele; Carrier-G = total of genotypes with the G allele; Non-Carrier-A = total of genotypes without the A allele; Non-Carrier-T = total of genotypes without the T allele; Non-Carrier-C = total of genotypes without the C allele; Non-Carrier-G = total of genotypes without the G allele.

**Table S5:** Association analyses among NLRP3 and CARD8 inflammasome haplotypes frequencies and risk/protection factors for coronary artery disease in SARS-CoV-2 infected individuals.

|  |  |  |  | | **Adjusted model** | |
| --- | --- | --- | --- | --- | --- | --- |
| **Genes SNP (rs)** |  | **Haplotypes** | **With coronary artery disease** | **Without coronary artery disease** |  | |
|  |  |  |  |  | **aOR^a^ (CI95%)** | ***P*-value^b^** |
|  | | CTGCC | 227 (24.1) | 8 (21.05) | Reference | |
|  |  | ACACC | 53 (5.63) | 2 (5.26) | 0.51 (0.03-8.97) | 0.646 |
|  |  | ACACG | 155 (16.45) | 5 (13.16) | 1.01 (0.24-4.16) | 0.989 |
|  |  | ACGCC | 23 (2.44) | 0 (0) | 0 (0-Inf) | 0.997 |
|  |  | ACGCG | 46 (4.88) | 3 (7.89) | 1.5 (0.29-7.63) | 0.627 |
| **NLRP3**  **rs1539019 rs4612666 rs3806268 rs35829419 rs10754558** | | ATGAG | 4 (0.42) | 0 (0) | 0 (0-Inf) | 0.999 |
|  |  | ATGCC | 62 (6.58) | 2 (5.26) | 0 (0-Inf) | 0.995 |
|  |  | ATGCG | 3 (0.32) | 0 (0) | 0 (0-Inf) | 0.999 |
|  |  | CCACC | 110 (11.68) | 4 (10.53) | 1.11 (0.21-5.78) | 0.903 |
|  | | CCACG | 31 (3.29) | 0 (0) | 0 (0-Inf) | 0.996 |
|  |  | CCGAC | 1 (0.11) | 0 (0) | 0.43 (0-Inf) | 1 |
|  |  | CCGCC | 135 (14.33) | 3 (7.89) | 0.66 (0.12-3.48) | 0.623 |
|  |  | CCGCG | 45 (4.78) | 2 (5.26) | 1.79 (0.18-18.18) | 0.622 |
|  |  | CTACC | 5 (0.53) | 1 (2.63) | 9.99 (0.71-140.45) | 0.088 |
|  |  | CTACG | 3 (0.32) | 1 (2.63) | 2.37 (0.08-72.29) | 0.620 |
|  |  | CTGAC | 2 (0.21) | 0 (0) | 0 (0-Inf) | 0.999 |
|  |  | CTGAG | 8 (0.85) | 2 (5.26) | 12.25 (0.69-217.99) | 0.088 |
|  |  | **CTGCG** | 29 (3.08) | 5 (13.16) | 11.82 (2.43-57.59) | **0.002** |
| **CARD8**  **rs2043211**  **rs6509365** | | AA | 664 (69.89) | 21 (55.26) | Reference | |
|  |  | AG | 37 (3.89) | 3 (7.89) | 1.93 (0.35-10.61) | 0.449 |
|  |  | TA | 2 (0.21) | 1 (2.63) | 32777812.85 (0-Inf) | 0.989 |
|  |  | TG | 247 (26) | 13 (34.21) | 1.51 (0.63-3.64) | 0.354 |

^a^Odds ratios were adjusted by skin color, schooling, gender, age, and associated comorbidities such as diabetes mellitus and obesity or previous bariatric disease; ^b^*P*-values were calculated using the unconditional logistic regression model. Associations were considered significant with a value of * *P* < 0.05. aOR: adjusted odds ratio; 95% CI: 95% confidence interval; NC: not calculated; N: number of individuals in each group.
